# Simulation-based skills training: a qualitative interview study exploring surgical trainees’ experience of stress

**DOI:** 10.1186/s41077-022-00231-2

**Published:** 2022-10-22

**Authors:** Maria Suong Tjønnås, Anita Das, Cecilie Våpenstad, Solveig Osborg Ose

**Affiliations:** 1 Department of Neuromedicine and Movement Science (INB), Faculty of Medicine and Health Sciences, NTNU, Norwegian University of Science and Technology, N-7491 Trondheim, Norway; 2 SINTEF Digital, Department of Health Research, SINTEF, P.O. Box 4760 Torgarden, NO-7465 Trondheim, Norway; 3 Department of Clinical and Molecular Medicine (IKOM), Faculty of Medicine and Health Sciences, NTNU, Norwegian University of Science and Technology, N-7491 Trondheim, Norway; 4 The national research center for Minimally invasive and Image-guided Diagnostics and Therapy (MiDT), St. Olavs Hospital, Trondheim University Hospital, Prinsesse Kristinas gate 5, Postbox 3250 Torgarden, NO-7006 Trondheim, Norway

**Keywords:** Simulation-based training, Stress experience, Surgical trainees, Laparoscopy, Box-trainers, VR simulators, Qualitative interview study

## Abstract

**Introduction:**

Stress can affect the ability to acquire technical skills. Simulation-based training (SBT) courses allow surgical trainees to train their technical skills away from stressful clinical environments. Trainees’ subjective experiences of stress during SBT courses on laparoscopic surgery remains understudied. Here, we explored the subjective stress experiences of surgical trainees during mandatory laparoscopic SBT courses. We aimed to obtain a broader understanding of which factors of the simulation training the trainees perceived as eliciting stress.

**Methods:**

A qualitative study with semistructured individual interviews was undertaken to explore trainees’ subjective experiences of stress. Twenty surgical trainees participated while attending courses at a national training center for advanced laparoscopic surgery. Questions explored trainees’ stress experiences during the SBT courses with a focus on perceived stressors related to laparoscopic simulation training on two box-trainers and one virtual reality simulator. Interview data were analyzed using inductive, qualitative content analysis methods to identify codes, categories, and themes.

**Results:**

Findings indicated that trainees have a variety of stress experiences during laparoscopic SBT. Three main themes were identified to be related to stress experiences: simulation task requirements, psychomotor skill levels and internal pressures, with subcategories such as task difficulty and time requirements, unrealistic haptic feedback and realism of graphics, inconsistent and poor technical performance, and self-imposed pressures and socio-evaluative threats.

**Conclusions:**

Insights into surgical trainees’ experience of stress during laparoscopic SBT courses showed that some stress experiences were directly related to simulation training, while others were of psychological nature. The technical and efficiency requirements of simulation tasks elicited stress experiences among trainees with less laparoscopic experience and lower levels of psychomotor skills. Self-imposed pressures played an integral part in how trainees mobilized and performed during the courses, suggesting that levels of stress might enhance laparoscopic simulation performance. For course facilitators aiming at optimizing future laparoscopic SBT courses, attending to the realism, providing clarity about learning objectives, and having awareness of individual differences among trainees’ technical level when designing the simulation tasks, would be beneficial. Equally important to the laparoscopic SBT is to create a psychological safe learning space in order to reduce the internal pressures of trainees.

**Supplementary Information:**

The online version contains supplementary material available at 10.1186/s41077-022-00231-2.

## Introduction

Surgical simulation-based training (SBT) allows surgical trainees to practice in an environment free of operating room specific stressors and without putting patients at risk [1]. In the field of laparoscopic surgery, simulators have long been utilized for practicing as adjuncts to the traditional master–apprentice model [2]. Simulators are used for training basic laparoscopic skills, as well as complex laparoscopic procedures [3–5]. Basic laparoscopic SBT typically involves the use of box-trainers and virtual reality (VR) simulators [6, 7]. To master laparoscopic techniques, trainees face a steep learning curve because of the intricate handling of stiff laparoscopic instruments, hand-eye coordination, bimanual efficiency, reduced sensation of touch, overcoming the Fulcrum effect (the tip of the surgical instrument moves in the opposite direction to the hand movement), and depth perception of three-dimensional (3D) surgical scene on two-dimensional (2D) images [8, 9]. With such demands on the learner’s psychomotor abilities, training in laparoscopic skills using simulators can induce both physiological and psychological stress responses [10–12].

Within the Lazarus and Folkman theoretical concept, the stress response is interpreted as a result of the person–environment interaction and arises when the demands of the person are not in balance with their perceived resources [13]. These demands can be of physical, cognitive, and social characteristics, and are often referred to as stressors [14–16]. How a person experiences and responds to stressors is dependent on their resources such as coping strategies, prior experience, personality traits, and their appraisal of socio-evaluative threats [17–20]. Therefore, they might differ from another person’s experience and response, even if the stressors or the situational settings are similar. Previous studies have shown that stress influences the acquisition of surgical skills, because excessive amount of stress can impair such performance, whereas low and moderate levels of stress might facilitate surgical performance [10–12, 21]. Despite substantial research on physiological stress responses related to laparoscopic skill acquisition in laboratory studies and the operating room [11, 22, 23], few studies have focused on trainees’ subjective experiences of stress during laparoscopic SBT courses. Studies using an interview-based approach, questionnaires, and self-reporting of stress have given some insights into the subjective stress experiences in simulation-based laboratory and interventional studies [12, 24, 25]. However, much remains unclear about which factors of laparoscopic SBT the trainees experience as stressful, in particular when performing simulation tasks in SBT course-based settings. An exploration into trainees’ subjective experiences would increase the understanding of how stress can influence simulation training and performance. Therefore, the aim of this study was to explore surgical trainees’ stress experiences associated with SBT of laparoscopic skills and performance.

Our research question was: “What subjective stress experiences do surgical trainees have related to simulation-based laparoscopic skills training and performance in an SBT course setting?”

## Methods

**Fig. 1:**
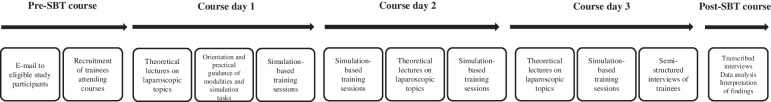
Schematic of the study design and timeline showing the distribution of the SBT sessions and topical lectures during the courses

A qualitative research approach was employed to explore surgical trainees’ subjective experiences of stress during laparoscopic SBT courses [26]. This method allowed us to explore trainees’ perspectives and thought processes, as well as nuances and variables underlying their stress experience when training on three different laparoscopy simulators. We used an interview-based method and conducted semistructured individual interviews with surgical trainees attending SBT courses [27, 28]. The study design and timeline are illustrated in Fig. 1. The interview data were analyzed using inductive qualitative content analysis, an approach that allows for systematic and reliable analysis of codes, categories, and themes [27, 29]. The main themes presented are the authors’ subjective interpretation of the information yielded from the categorization and abstraction process of the interview data [30]. The study was approved by the Regional Committees for Medical and Health Research Ethics of Norway (Reference number: 2018/2414) and followed the Consolidated Criteria for Reporting Qualitative Research (COREQ) reporting guideline (Additional file 1).

### The interview guide

The interview guide (Additional file 2) was developed based on the literature on the topic, and on information from former trainees and surgeon instructors. A pilot study with four trainees was carried out to test and revise the interview guide. We anticipated the trainees’ variations in the perception and interpretation of the term *stress*; however, to encourage trainees to talk without constraints about the topic, we decided not to present a specific concept of stress ahead of the interviews.

### Sampling

Participants were included through purposive sampling. We recruited surgical trainees who were enrolled in courses at a national training center for advanced laparoscopic surgery. The trainees were in their first, second, or third year of their surgical specialization, and from different hospitals across Norway. The trainees were invited by e-mail to join the study and additional recruitments were made during the courses.

### Demographics

Participants’ demographics were collected in the introduction part of the semistructured interviews. Information about gender, age, prior work experience, laparoscopic surgery experience, and simulation experience were collected.

### Setting and context

The laparoscopic SBT was performed as part of a mandatory course on basic laparoscopic skills for surgical trainees in Norway. The course program consisted of several lectures followed by standardized laparoscopic SBT sessions (Fig. 1; Table 1). Each course lasted for three consecutive days and was rounded off with a written exam. These courses are normally taken early in the surgical specialist’s education (on average 5 years of education in Norway), and no previous formal laparoscopic training was required of the trainees. The trainees admitted to the courses had never taken them previously. The courses are mandatory once during the education.

**Table 1:** Description of the simulator type, pre-simulation training preparation/briefing, simulation-based training, and feedback/debriefing

**Simulator type**			
Simulator make and model^a^	Box-trainer (D-box, Covidien Surgical Box, Mansfield, MA, USA).	Box-trainer (Pulsatile organ perfusion (P.O.P) box-trainer, Optimist, Innsbruck, Austria).	VR-simulator (LapMentor™, 3D systems, Littleton, USA).
Simulator functionality^a^	The D-box, a box-trainer had a fixed video camera and a light source inside the box, and entry holes for the laparoscopic instruments. Laparoscopic graspers were used for the simulation tasks. The box-trainer offered training of bi-manual dexterity, interpretation of 3D images on a 2D screen, depth perception, and hand-eye coordination. Limitations: the light source was fixed in one angle which could impact the visibility.	The P.O.P-trainer, a box-trainer which provided training on artificial models and real tissue using authentic laparoscopic instruments. The simulator had a top lid with entry ports for trocars. Both artificial organ models and porcine tissue were used to facilitate the simulation tasks. Limitations: the pulsating function which simulates circulation of liquids in real tissue was not available.	The LapMentor™, a VR simulator provided training in a user interface with 3D data-generated images that offered a visually real-life experience. The simulator had built-in haptic technology. The module for basic training was set for exercises with pegs, appendectomy, and cholecystectomy. Limitations: the VR simulator software would only accept a specific way of performing a procedure.
**Pre-simulation training preparation/briefing**			
Participants orientation to the simulator ahead of the training sessions^a^	Trainees were given outline of the simulation training task and the requirements, and practical guide in using the simulator technology.	Trainees were given outline of the simulation training task and the requirements, and practical guide in using the simulator technology, and instructions on how to work in pairs.	Trainees were given outline of the simulation training task and the requirements, and practical guide in using the simulator technology, which included software and hardware.
Participant orientation to the environment ahead of the training session^a^	Trainees were assigned to one of six simulators, which were placed on bench tops, adjusted to trainees’ heights. Instructors were available on site, and trainees were allowed to engage in conversations or observations during training.	Trainees were assigned to one of five simulators, which were placed on bench tops with the associated computer screen and laparoscopic equipment mounted on a rack. Instructors were available on site, and trainees were allowed to engage in conversations or observations during training.	Trainees were assigned to one of two VR simulators. The VR simulator system was a self-contained unit with laparoscopic instruments and computer screens. Instructors were next to the trainees during training. Trainees were allowed to engage in conversations or observations during training.
Group vs. individual practice^a^	The trainees performed the simulation tasks alone.	The trainees performed the simulation task in pairs.	The trainees performed the simulation tasks alone.
**The simulation-based training**			
The simulation-training task	Pre-simulation training: Consisted of exercises to familiarize the trainee to the graspers, to using both hands, and the depth perception. Main simulation-training task: Passing a thread through multiple loops in a pre-set path.	Pre-simulation training: Consisted of exercises to familiarize the trainee to the hand-eye coordination and depth perception, and suture exercises on artificial model organs. Main simulation-training task: Suture and knot-tying exercises on porcine tissue.	Pre-simulation training: Consisted of exercises to familiarize the trainee to the VR interface, i.e., peg transfers. Main simulation-training task: Full cholecystectomy and appendectomy procedures.
Learning objectives^a^	(1) Be familiarized with the laparoscopic instruments, practicing of bi-manual dexterity, training of hand-eye coordination, and to be able to interpret depth on the PC screen. (2) Pass a thread through multiple loops in a pre-set path.	(1) Be familiarized with the laparoscopic instruments, and to be able to interpret depth on the PC screen. (2) Practicing suture and knot-tying techniques. (3) Accomplishing a surgical square knot.	(1) Be familiarized with the software and hardware of the simulator. (2) Accomplishing a peg transfer simulation task. (3) Accomplishing an appendectomy and a cholecystectomy.
Requirements/assessment^a^	Accomplishing the main simulation-training task within 1.5 min.	Accomplishing the main simulation-training task within 2.5 min.	Assessment of errors, time to complete task and other assessment metrics were logged in the program.
Frequency/repetitions^a^	No restrictions on number of repetitions.	No restrictions on number of repetitions.	No restrictions on number of repetitions.
**Feedback/debriefing**			
Feedback^a^	Instructors gave feedback on the technical performance and provided guidance on performance if needed.	Instructors gave feedback on technical performance and provided guidance on performance if needed.	Instructors gave feedback on technical performance and provided guidance on performance. Computer program provided text-based feedback on errors, time to complete task, and other metrics.
Instructor presence^a^	One instructor was present throughout all simulation sessions.	Two to three instructors were present throughout all simulation sessions.	One instructor was present throughout all simulation sessions.
Instructor characteristics^a^	Instructors were medical educators with more than 10 years of experience. The main instructor held a PhD in medical technology with specialization in laparoscopic simulation.	Instructors were medical educators with more than 10 years of experience. Instructors were general surgeons with specialization in gastroenterology and gynecology, with 25–30 years of laparoscopic experience.	Instructors were medical educators with more than 10 years of experience. Instructors were general surgeons with specialization in gastroenterology, with 25–30 years of laparoscopic experience.
Non-simulation interventions and adjuncts^a^	Lectures on laparoscopic topics, laparoscopic techniques and learning outcomes of the box-trainers and VR-simulator were given prior to the simulation-training sessions on each course day.		

^a^Elements from Cheng et al. (2016) Table 3 Key Elements to Report for Simulation-Based Research [31]

### Description of the laparoscopic SBT

Before the SBT sessions, the trainees were given orientation to each simulator, simulation tasks, and task requirements by instructors (detailed descriptions are provided in Table 1). Three different simulator modalities were used, two box-trainers (D-box, Covidien Surgical Box, Mansfield, MA, USA, and P.O.P-trainer, Optimist, Innsbruck, Austria) and one VR simulator (LapMentor™, 3D Systems, Littleton, CO, USA). The trainees trained for 1 h on each simulator before rotating to the next simulator, and the simulation sessions lasted for 3 h on the first day, 5 h on the second day, and 3 h on the last day. Trainees operated alone on one of the box-trainers and the VR simulator, while the trainees were paired on the other box-trainer. The instructors provided guidance and feedback on performance and technique throughout all training sessions.

### Data collection

Data were collected from March 2019 to September 2021. The trainees were interviewed at the course facilities shortly after completion of the simulation training sessions. The primary author (MST) conducted all interviews. MST, an experienced interviewer, had training in conducting semistructured interviews with clinical personnel. MST was not involved as an instructor during the courses, and did not have any influence on trainees’ training or performances. The interviews lasted for 20–60 min. The interviews included follow-up questions to clarify statements and explorations into relatable themes. To ensure the validity of the data, each interview was summarized and validated orally by trainees regarding the content, accuracy, and meaning of statements. Interviews were consecutively transcribed and when preliminary analysis of the interviews showed that no new themes relating to the main topic were provided by new interviews, it was consensus that sufficient data had been obtained to answer the research question [32], and the data collection ended. All interviews were audio recorded using the dictation machine (Olympus WS-852) and transcribed verbatim by MST. Management and storing of audio recordings were in accordance with the regulations of the Regional Committees for Medical and Health Research Ethics of Norway.

### Analysis

In the inductive, qualitative contents analysis process adapted from Graneheim and Lundman [27], all transcripts were reviewed, and the meaning units (all statements and sentences related to trainees’ stress experiences associated with training, performance, and course settings) were identified, condensed, and inductively coded by author MST in collaboration with author SOO. The coded interviews were read and discussed by the authors, and a consensus on codes was reached eventually. The codes were sorted into categories and further subjected to an abstraction process for the identification of main themes. In this study we identified main themes as the general stress experiences of the study group, leaving out the stress experiences only reported by one participant. The identification of main themes required an interpretation process of data involving repeated sessions of discussions involving reflection, comparison, and recategorization of themes. Table 2 lists the main themes, sub-categories, and meaning units (with example excerpts). The interpretation process yielded three main themes with associated sub-categories. Themes were validated by rechecking the transcripts to ensure they were derived from the original data. The method described by Ose [33] was employed to manage the data.

**Table 2:** Themes, sub-categories, and meaning units/excerpts

Themes	Meaning units/excerpts
**Theme 1: Stress experiences related to simulation task requirements**	
**Sub-categories**	
Simulation task efficiency	“When we were just trying out things (box-trainers), I felt it went well and I did great! But as soon as we started timing ourselves, it suddenly went horribly!” (ST10).
	“Yes, that made me stressed (the P.O.P-trainer)! Once you start the stopwatch, you’ll just get more stressed.” (ST13).
	“It’s a basic task which should be quite simple and straightforward, but then you add the time aspect, which makes it stressful.” (ST14).
	“When you have a goal (time requirements), then it automatically becomes a little more interesting.” (ST14).
	“I don’t think I did well because I spent a lot of time on the tasks. At first, I couldn’t handle the time pressure and I didn’t manage to fulfil the time requirements right away.” (ST19).
	“When I started the stopwatch the first day, I got such an overwhelming feeling of competition, it was really strange, and I just turned up the speed and I performed fast. It was a really encouraging feeling and just fun. It was a very positive experience. But eventually I think the time pressure became very draining, so I stopped timing myself.” (ST20).
	“The most stressful simulation training task is the D-box. It’s probably because you are aware of the time requirements; it’s such a narrow time requirement, you are barely within the requirements every time.” (ST4).
	“But the time requirements were just… so… the time pressure ruined the whole course for me… he he he…” (ST10).
Simulation task difficulty	“I think I spent too much time on the parts of the simulation task where I had to suture. I struggled with positioning the needle correctly when I was using the needle holder, so that frustrated me quite a bit. When I felt I had spent too much time on this task, I got a little stressed.” (ST8).
	“Those grasper entry holes were really small, and the graspers were of poor quality (laughing), and then they didn’t really act the way I wanted them to! I think the time factor had an impact. As soon as you have to compete against the clock, everything gets worse ...” (ST14).
	“The surgical knot was difficult to handle anyways, so when we started timing ourselves, it made me spend even more time getting the knot right… I felt that was stressful!” (ST3).
	“I thought the box-trainer was really challenging and it took me a very long time before I managed to crack the code on how to do it… just to figure how to get through the loops without having those awkward choppy movements.” (ST16).
	“Yesterday I didn’t do so well so I had to take a little break. I needed to figure out how to do the tasks correctly. I then focused on completing them accurately instead of doing them as fast as I could. The more accurately I performed, the faster I finished the tasks. I felt less stressed when I figured this out.” (ST20).
Unrealistic haptic feedback and lack of realism in the graphics	“You don’t get the same touch sensation from the VR tissues. You discover the computer image resolution is not that great, because you might think that you grabbed something, but you really didn’t...the VR image is deceiving you a bit.” (ST2).
	“Considering what you can get on a regular gaming PC nowadays, comparatively this VR simulator has basic graphics, there is no doubt about that.” (ST14).
	“The VR simulator is getting better, but so far the graphics are not sufficiently realistic and the VR operation scene does not perform as it would in real life.” (ST12).
	“When the appendix does not want to get into the endo-bag because it does not understand gravity, then the whole thing becomes a bit too unrealistic…Yes, and it fails to mimic crucial things like tissue…” (ST16).
	“My experience on the VR simulator is that it is unrealistic. In reality, the operation scene will never look like that. Additionally, I can only perform the procedure the way the simulator directs, and I can only use certain instruments that are predetermined by the computer program; thus, it all feels a bit fake, because those instruments are not the same ones that we use at our workplace.” (ST16).
Minimal stress related to VR simulation tasks	“It (VR simulator) wasn’t stressful at all, I thought it was fun!” (ST10).
	“The issue is that the tissue you are trying to grab does not behave as it would in a real patient; this shatters the illusion of an operation. The failure is due to how the tissue moves and reacts, more so than the overall graphics.” (ST11).
	“It’s a lot like in a computer game.” (ST2).
Impact on motivation	“I think it isn’t sufficiently sophisticated to really challenge you like a proper surgery would. Sure, you work through the steps of a surgical procedure, and you can gain an understanding of where the organs are located. But to be honest, I wouldn’t be bothered to spend a lot of time practicing on a mediocre virtual reality experience.” (ST5).
	“I always exceeded the time requirements slightly. Maybe it’s because it didn’t feel very realistic, it felt more like a game.” (ST19).
**Theme 2: Stress experiences related to psychomotor skills level**	
Low technical performance	“I felt stressed and frustrated when training on the D-box, because in the beginning I struggled with the technique and to achieve the results I felt I should have obtained. So, I became quite frustrated, which caused me to perform worse and then I got even more stressed, and it became a downward spiral.” (ST8).
	“I felt that I didn’t handle the technicalities so well (on box-trainers).” (ST15).
	“It was challenging (laparoscopic knot-tying), I spent a long time without realizing what I was doing wrong, and that was very frustrating. It just didn’t work! It was tough when I felt I had tried really hard and still did not complete the task requirements. I tried everything the instructors suggested, and it still didn’t work, and then I began to get tired, and it all became even more difficult.” (ST19).
Inconsistent performance	“I’m frustrated that the instrument and the thread (of the D-box) do not move in the direction I want them to! It’s frustrating that sometimes it works for me, while other times it doesn’t; it’s hard to figure out what you do correctly or why you are failing at something.” (ST2).
	“Yes, I’m stressed, I am actually doubting if I will master the requirements even though I have trained a lot on box-trainers previously. I’ve not had the opportunity to practice at my hospital recently, as all the box-trainers there are broken. It’s a specific simulation task. I’m sure that if you have not been able to practice prior to the course it’s difficult to meet the requirements. Obviously, it helps to do specific training.” (ST20).
Lack of laparoscopic training	“The simulation tasks stressed me because I’ve never tried this kind of simulator (VR simulator) before. I’ve not performed much surgery yet, and suddenly I had to deal with anatomy too, and it was excruciating because I was so scared of doing something wrong. In a real surgery I would have required very detailed guidance throughout the procedure.” (ST20).
	“At our workplace, it is very uncommon for senior surgeons to suture laparoscopically, and for surgical trainees to do that, it just doesn’t happen.” (ST9).
	“I have not sutured laparoscopically prior to this course, so the P.O.P-trainer was a little challenging.” (ST14).
	“I spent a lot of time at the start of the training session just figuring out how to change the equipment and other stuff.” (ST1).
	“I was a bit annoyed with the appendectomy, because it is a procedure I don’t have much experience with, and I also got a little frustrated because I realized while I’m doing the simulation task that this is probably not how it is done in a real surgery.” (ST6).
**Theme 3: Stress experiences related to internal pressures**	
Self-imposed pressures	“No, yes, I was frustrated and annoyed by how bad I performed just now… I managed to finish under two minutes a couple of times, but now I relapsed completely to my starting levels again, timewise.” (ST9).
	“As soon as we started with the training, even without timing the task, I was already stressed.” (ST13).
	“I was slow in the beginning and that was stressful. You also stress the moment you start trying to beat your own times.” (ST15).
	“Yes, you put pressure on yourself and then it becomes more stressful.” (ST18).
	“I felt that I did better yesterday, but now it is getting worse and I felt more stressed…” (ST15).
	“I consider myself as a pragmatic person; I expected to perform well on these tasks, and I managed to master the tasks quite fast, but all of the sudden I started to get really stressed.” (ST20).
	“It’s so intense, you want to beat your own record, and when that doesn’t happen, you become overwhelmed… but I did not perceive the situation as stressful; tense is more befitting.” (ST13).
Socio-evaluative threat	“I’m stressing with the time requirements. I want to keep improving my results. This D-box is a classic throughout the country, just about every hospital has one. So, I want to achieve excellent results.” (ST4).
	“Today has been stressful, because we tried to break our personal records and then the instructors came to observe when it was going pretty well, and then all of the sudden it went downhill…” (ST15).
	“I expected more from myself on the P.O.P-trainer because it simulates exactly what I should master in a real work situation.” (ST20).
	“I noticed that I became stressed when I realized I was slower than the person next to me. I was quite displeased, while thinking his results were a little undeserving (laughing). I noticed how easy the simulation task was for him, so I became really consumed with comparing myself to him.” (ST20).
	“It’s rather stress from the environment and the circumstances that affects me, more so than the simulation exercise itself.” (ST13).
	“I don’t like the time pressure, I’m not really a competitive person.” (ST1).
	“I stress myself; I do well with communication but I’m bad at practical stuff.” (ST13).

## Results

Three main themes of stress experiences were identified through analysis. These related to (1) simulation task requirements such as task difficulty and time requirements, in addition, themes related to haptic feedback and realism of graphics; (2) psychomotor skill levels including inconsistent and poor technical performance; and (3) internal and self-imposed pressures, and socio-evaluative threats. These are listed in Table 2.

### Participants

This study contained 20 surgical trainees. Their ages ranged from 28 to 42 years (median 34) and were of both genders with 55% identifying as female. The study participants were heterogeneous regarding prior laparoscopic experience as surgical trainees and in terms of simulation training experience as shown in Table 3. Two trainees had prior experience with the VR simulator, another two had substantial experience with the box-trainer (D-box); otherwise, the trainees had little experience with the simulators.

**Table 3:** Study participants’ prior work experience and simulator training experience

Participants (***n*** = 20)	Work experience as surgical trainees (months)	Training experience in laparoscopic techniques on box-trainers, VR-simulators, or robotic simulators (hours)
ST1	2	16
ST2	7	3
ST3	9	2
ST4	1	30
ST5	2	50
ST6	7	20
ST7	6	0
ST8	2	10
ST9	2	30
ST10	6	4
ST11	13	3
ST12	12	1
ST13	24	60
ST14	30	2
ST15	32	4
ST16	3	15
ST17	24	0
ST18	24	20
ST19	9	3
ST20	12	0

Participants of the study are identified as ST1, ST2, ST3, … ST20

### Stress experiences related to the complexity of simulation tasks

Trainees reported feeling stressed by the time requirements set by the box-trainers because they felt these were too rigid, and the fear of not meeting these requirements triggered stress and caused impaired performances in the early stages of training. In particular, trainees with less laparoscopic experience and those using unfamiliar laparoscopic equipment reported that the time requirements added an extra layer of stress to the simulation training, which led to poor performances. Conversely, some trainees reported that the stress triggered by the time requirements motivated them to intensify their training during the course and helped them to achieve better performance results. On the other hand, trainees with previous experience with box-trainers reported experiencing minimal stress related to training on these simulators, which was reflected in the faster accomplishment of task requirements and higher procedural efficiency. Overall, trainees reported that the stress they experienced early in the simulation course heavily affected their motivation and performance results during the course. However, all study participants passed the task requirements eventually, regardless of their initial stress levels. In contrast to the simulation training on the box-trainers, trainees reported experiencing minimal stress when training on the VR simulator. Trainees explained that they did not perceive the VR simulation tasks as “real enough” to make them feel stressed. Trainees repeatedly stated that they found the haptic feedback of the VR simulator to be too unrealistic and left them unable to differentiate between the different tissues represented in the VR operating scene, which was claimed as a major source of their demotivation toward further practice with this modality. Illustrative excerpts are listed in Table 2.

### Stress experiences related to motor skills level

Trainees reported experiencing stress from their perception of poor technical performance. They expressed stress and frustration at not performing to the technical level that was required, describing stress levels as especially high when not being able to perform consistently or at technically high levels on every session on the simulators. The trainees with little previous experience with laparoscopic simulators or techniques expressed how this was reflected in their performance. The combination of handling unfamiliar laparoscopic instruments and performing demanding simulation tasks was described to have had a negative additive effect on performance and increased trainees’ stress experiences. Illustrative excerpts are listed in Table 2.

### Stress experiences related to internal pressures

Trainees described how they felt stressed by the internal pressures of performing well on the simulators. On reflection, the trainees admitted that these internal pressures were their own self-imposed need to perform at high levels. This manifested as the constant need to improve upon their performance results, even if they already had accomplished the task requirements. The pressure was described as most prominent when training on the box-trainers, in particular the D-box. Several trainees reported experiencing stress when they found themselves comparing their results with those of other course participants. For other trainees, the comparison was seen as beneficial for their motivation toward performance objectives because it prompted them to engage in more intensive training, and ultimately resulting in improved performance. Illustrative excerpts are listed in Table 2.

## Discussion

Our aim was to explore surgical trainees’ stress experiences during laparoscopic SBT courses to obtain a broader understanding of which factors of the SBT the trainees perceived as eliciting stress. The trainees experienced a variety of stressors, including some that are directly related to simulation training, such as the simulation task requirements and trainees’ psychomotor skills, alongside those of a psychological nature, such as internal pressures. The specific themes identified included task difficulty, time requirements, unrealistic haptic feedback and realism of graphics, inconsistent and low technical performance, self-imposed pressures, and socio-evaluative threats. The study also revealed that trainees have minimal stress experiences related to training on VR simulation tasks.

### Threats, challenges, and previous experience

The trainees viewed the time pressure of the simulation tasks as a major stressor, eliciting stress experiences that they reported as leading to an impaired or poor technical performance in the early stages of the simulation training. In surgical training, it is crucial for trainees to acquire high degrees of technical as well as procedural efficiency [8]. The simulation task requirements of the SBT course were designed to measure both technical performance and procedural efficiency, where efficiency was measured through time requirements (time to complete a given task). The findings suggest that with the pressure of specific time requirements, the technical performance suffered. Research has shown that novice surgeons, when faced with competing cognitively demanding tasks, may attend to one of the tasks at the expense of others [34]. Moorthy et al. showed an adverse effect of multiple stressors, including time pressure, on the performance of a simple laparoscopic transfer task. In particular, skill-based errors appear to increase under the effect of the stressors [35]. When not being able to complete the simulation tasks as required, the trainees expressed how they perceived the simulation tasks as threats. When an individual is faced with a task that is cognitively appraised as a threat, and the demands of the threat outweigh the individual’s perceived available resources to meet the situation, the result is distress, which can impair technical performance [13, 36, 37]. Our findings are consistent with the literature, where task difficulty and time pressure are described as major stress factors impacting the technical performance of trainees in the laboratory and in clinical settings [10, 38, 39]. Likewise, in other medical simulation fields, similar findings have been reported of the impact of stress on simulation training performances [21]. In a study by LeBlanc et al. using patient simulators, impairments in technical skill performance were observed in participants following stressful simulated scenarios [40]. However, for some of the trainees, the same simulation task stressors helped to improve their performance results by prompting them to intensify their training during the course. When an individual appraises a task as a challenge, as opposed to a threat, and the demand of the task is perceived to be within their capability of handling, the stress experience can induce motivation and focus their attention on improved performance [36, 37]. Previous studies have demonstrated that participants improved their technical performance when stressors were added to the simulation session [41]. In a study by Moawad et al., gynecology residents performed with higher efficiency when tested for laparoscopic skills under stressful conditions compared with the same conditions without stressors [42]. A couple of trainees in our study had substantial prior experience with box-trainers and reported experiencing minimal stress related to the task requirements. This suggests that previous experience with similar stressful situations might have modified their stress experience. This is supported by previous research finding that a person’s prior experience with similar stressful situations or stressors can modulate their stress response [19, 43]. In a study by Crewther et al., it was demonstrated that a 3-week simulation-based laparoscopic skills training program promoted stress adaptations, which supported the acquisition of performance skills in novice surgeons [44], suggesting that repeated exposure to stressors can even have beneficial effects on the acquisition of skills.

### Haptic feedback, realism of graphics, and motivation

Unlike training on the box-trainers, the trainees reported experiencing minimal stress during training on VR simulation tasks. The participants found the haptic feedback and the VR simulated tissue to be too unrealistic to stimulate a stress response during the simulation training, and some trainees expressed their demotivation toward further training on the VR simulator because of these limitations. This suggests that with the unrealistic haptic feedback and lack of realism in the graphics, the challenge of the surgical task failed, and thus diminished trainees’ stress experience and learner engagement [45, 46]. The role of haptic feedback and tissue realism of VR simulators have been discussed previously, showing that these are important features of the total simulation experience [47, 48]. However, the impacts of haptic feedback and realism on training and performance outcomes are variable. When the level of realism in a simulation task does not match the training objectives, apprehensions about the simulation modality as a training tool can develop, leading to resistance to training on these models [49–51]. Our findings are in line with the review by Gostlow et al. about voluntary participation in SBT of laparoscopic skills. The review showed that realistic haptic feedback and realism were important motivators for trainees to engage in repeated training activities on VR simulators [52].

### Inconsistent performance and low psychomotor skills

Here, the trainees emphasized how they felt stressed by not performing consistently at high technical levels on the simulators. As novice surgeons, the trainees might have been unaware of how technically complex and steep is the learning curve for mastering laparoscopic skills. To master these skills demands high psychomotor abilities that are improved by extended and repetitive training [8]. Fitts and Posner’s model of the acquisition of motor skills describes three successive phases (cognitive, associative, and autonomous) that learners need to go through when developing new motor skills [53]. To reach the autonomous phase—where skills become automatic—requires repetitive practice over a period [54]. Without the requisite extensive training to master this surgical technique, inexperienced trainees will not be able to perform at a high level or consistently on laparoscopy simulators [55]. Depending on the trainees’ previous laparoscopic training, the simulation task difficulty levels might not have been aligned with the level of their psychomotor skills, subsequently eliciting stress experiences in those trainees with lower psychomotor skill levels or little laparoscopic training, suggesting that the demands of the simulation tasks outweighed trainees’ resources in psychomotor skills [13]. In a simulation study by Wetzel et al., junior surgeons reported that technical challenges and the lack of competence acted as sources of stress during simulated surgical procedures. In the same study, it was demonstrated that experienced surgeons, despite having high stress levels during the simulated procedures, did not show deterioration in performance. Thus, experience and surgical competence can compensate for the undesirable effects of stress on technical performance [18].

### Self-imposed pressures

Trainees emphasized how important it was to perform well on the box-trainers because it was seen as basic to the SBT, and the self-imposed pressure to master these simulation tasks caused them to experience stress. The tasks performed on these simulators were designed to train basic bimanual dexterity, hand-eye coordination, and depth interpretation of 2D images, three essential skills surgeons need to master when performing laparoscopic procedures [8, 56]. In a practical simulation course setting, demonstration of technical skills is needed, with the risk of revealing weaknesses and proficiency gaps to peers and instructors, and consequently eliciting stress in trainees. Our findings suggest that trainees were worried that their performance results on the box-trainers could have been viewed as an indication of their skills, which could then be further interpreted as a reflection of their limited surgical competence [57]. Previous studies have shown that, when acting as a surgical trainee, mastering, and demonstrating basic technical skills is an important part of the process of developing surgical competence [2, 57, 58]. Trainees’ worries about acquiring sufficient surgical competence appear to be a concern that starts early in their surgical training and persist through to graduation [57].

### Socio-evaluative threats

Trainees described how they felt stressed when they compared themselves with other course participants during the training, prompting them to intensify their training to improve the results, even though they had already accomplished the requirements. Many of the trainees already had some experience in training on box-trainer simulators before the course. This created a large range in skills between those familiar or unfamiliar with the simulator before the course. Previous research has shown that variations in laparoscopic skills among trainees are dependent on their access to laparoscopic procedures, equipment, and practice time [59]. Our findings suggest that when the trainees performed in a course setting among peers and instructor observers, they might have perceived the social-evaluative threat of not performing on the same level as their peers to be high, causing them to experience stress [60]. However, in contrast to the time requirements and task difficulty, socio-evaluative threats provided positive motivation for training during the course, and subsequently an increase in performance. The findings suggest that the stress experience elicited by the social setting of the SBT courses may be beneficial to training and enhance trainees’ performance results. Our findings are in line with the study by LeBlanc et al. who showed that junior surgery residents—while experiencing moderate stress levels from the examination—improved their technical performance during simulation [61].

### Implications and future work

For trainees, the awareness of their stress response and how it influences their training process and performance during laparoscopic SBT courses might give them an indication as to what they might find stressful when performing laparoscopic techniques in real-life settings. By recognizing their stress-related limitations, trainees could use this as a signpost to initiate conversations with instructors or mentors about recognizing and managing stress in surgical training. Educators could encourage trainees to seek stress management training proactively in addition to extended psychomotor skills training, and trainees could start developing the coping strategies required for their professional role as surgeons [25, 62]. A broader understanding of trainees’ subjective stress experiences can aid in the development of SBT courses aimed at the acquisition of surgical skills. Educators could attain a clearer understanding of what aspects of different simulation tasks and simulator modalities are perceived as stressful, and how this influences trainees’ training process, and use this insight to adapt the simulation training. Adaptations such as adjusting the level of task difficulty to the proficiency of the trainees by considering the trainees’ level of experience and psychomotor skill, and their ability to cope with the challenges of the simulation tasks. Furthermore, reducing the simulation task into manageable steps, such as allowing the trainees to practice until they feel comfortable with the tasks before introducing the added pressure of time and other technical requirements, could improve trainees’ training process. To reduce the psychological stress pressures, instructors could clarify the learning objectives of the SBT tasks, and addressing the role of instructors and other observers attending the courses, which will set a psychological safe space for trainees to train in [63]. The relationship between the level of realism (i.e., fidelity) of the simulators, stress response, and learning outcomes is unclear [64–66]. Our findings indicate that higher levels of both structural and functional fidelity in VR simulation tasks might be beneficial. In particular, realistic haptic feedback appears essential to elicit stress experiences and thereby increase learner engagement and motivation [49]. Innovations within VR technology (i.e., immersive VR and augmented reality), might provide an optimal level of realism conducive to eliciting stress and facilitating the SBT experience [67]. Future studies exploring these themes would be useful for the development of VR simulation as a laparoscopic training tool.

### Limitations

This study had several limitations. Major parts of this study was conducted during the global COVID-19 pandemic which involved several challenges. The pandemic involved increased clinical demands in hospital units across the country, multiple quarantines, travel ban for clinicians and therefore cancellation of planned SBT courses, and comprehensive COVID-testing pre-course admission. However, several aspects of the SBT remained as before: The SBT course settings, the learning objectives, the instructors and procedures. This preserved the integrity of the SBT course. For the study, it involved an extended time period for data collection; however, we do not consider that the extended time span had impact on the study findings. COVID-related adjustments during the data collection included the requirement to keep distance (1.5–2 m) and the need to wear facemasks during the interviews. This made it more challenging for the interviewer to identify non-verbal facial expressions, which may give cues for the interviewer in understanding if and when it is appropriate to go forward with more probing question. Considering the semi-structured form of the interviews, we do not think that this influenced the topics discussed and the findings as thus. At the risk of being interpreted as weak, or vulnerable to stress, trainees might have been guarded in their answers regarding stress-related themes. We ensured anonymity (de-identified audio recordings of interviews and transcripts) and chose to conduct one-on-one interviews as opposed to group interviews, but we do not know whether the trainees felt safe enough to disclose all their stress experiences to us. Therefore, more time to establish adequate levels of trust would be required for trainees to feel safe enough to be able to speak freely [28]. Trainees’ individual laparoscopic experience before the SBT course might have influenced their decision to join the study, with the risk of self-selecting bias. However, we sought a broad understanding of how stress is experienced by trainees during the SBT courses, which also included trainees with extensive prior laparoscopic experience. Despite the risk of self-selecting bias, the trainees represented a realistic sample. In this article, we did not include instructor’s perspectives on how stressful experiences might impact simulation training because we consider it to be outside the overall aims. However, the instructor’s role in a simulation training setting might have an impact on participants’ performance, so further research should elaborate on this topic [22, 68]. The generalizability of findings in qualitative studies is limited because of the nature of the findings being the subjective experience of the trainees; however, we believe our findings provide valuable insights into plausible perceptions and reactions from trainees attending laparoscopic SBT courses.

## Conclusion

Insights into trainees’ stress experiences during laparoscopic SBT courses revealed how stress can both enhance and diminish performance, and influence trainees’ motivation for training during the course. This illustrates the powerful impact of stress experiences on the acquisition of surgical skills. In this study, the difficulty of simulation tasks, psychomotor skill levels, and trainees’ self-imposed pressures were identified as key factors in their subjective stress experiences. Overall, trainees had varied stress experiences depending on the perceived difficulty level and the realism of the simulation task, with previous simulator experiences modifying the stress experience. Efficiency requirements and technical difficulties of the simulation tasks elicited stress experiences among trainees with less laparoscopic experience and lower psychomotor skill levels. Self-imposed pressures and socio-evaluative threats appear to have an integral part in how trainees mobilized and performed during the courses, with levels of stress enhancing their performance. For course facilitators aiming at optimizing future laparoscopic SBT courses, attending to the realism of the simulation tasks, providing clarity about learning objectives of the simulation tasks, and having awareness of individual differences among trainees’ technical level when designing the simulation tasks, would be beneficial for the training process. Equally important to laparoscopic SBT is to create a psychological safe learning space for trainees in order to reduce their internal pressures.

## Supplementary Information

**Additional file 1: Table S1.** Report in accordance with the COREQ guidelines-checklist for reporting qualitative research. Description of data: Completed checklist in accordance with the COREQ guidelines.

**Additional file 2.** Interview guide. Description of data: Topical questions used in the interviews for data collection.

## Data Availability

The datasets used or analyzed during the current study are available from the corresponding author on reasonable request. The data set is in Norwegian.

## Abbreviations

COREQ: Consolidated Criteria for Reporting Qualitative Research
P.O.P-trainer: Pulsatile organ perfusion trainer
SBT: Simulation-based training
VR: Virtual reality
