# Supplementary material for: Simulation-based skills training: a qualitative interview study exploring surgical trainees’ experience of stress
Source: Adv Simul (Lond). 2022 Oct 22;7:33. doi: 10.1186/s41077-022-00231-2 (PMC9588224; doi:10.1186/s41077-022-00231-2)
Supplement: Supplementary file 1 — Additional file 1: Table S1. Report in accordance with the COREQ guidelines-checklist for reporting qualitative research. Description of data: Completed checklist in accordance with the COREQ guidelines. [file 41077_2022_231_MOESM1_ESM.docx]

**Table S1. Report in accordance with the COREQ guidelines-checklist for reporting qualitative research.**

| No item | Description |
| --- | --- |
| *Domain 1: Research team and reflexivity* |  |
| Personal characteristics |  |
| 1. Interviewer/facilitator | MST conducted all interviews. |
| 2. Credentials | The first author and scientific researcher MST, was a PhD student, holds a MSc. in Physiology, is a registered nurse (RN), with research experience of more than 10 years; AD and CV both hold a PhD in Medical Technology and work as associate professors at the Norwegian University of Science and Technology in Trondheim, Norway; SOO holds a PhD and is a senior researcher with more than 15 years of research experience in health care services and socioeconomics. |
| 3. Occupation | MST is a scientific researcher at SINTEF and PhD student at the Norwegian University of Science and Technology in Trondheim, Norway. |
| 4. Gender | MST, AD, CV, and SOO are all female. |
| 5. Experience and training | MST had experience in research from different areas including quantitative and qualitative research, with more than 10 years within the physiological research field, and more than 5 years of clinical experience. |
| Relationship with participants |  |
| 6. Relationship established | There was no relationship between researcher and the participants. The researcher and the participants had never met prior to the courses, and there were no dependence issues between researcher and participants. |
| 7. Participant knowledge of the interviewer | Participants were given information about the researcher and study goals through written information about the project ahead of the courses. The participants who asked for more information, were given additional information. |
| 8. Interviewer characteristics | The article includes information about the professional background of the interviewer. The main interest of MST in the topic was grounded in her PhD studies on stress and laparoscopic simulation-based training. |
| *Domain 2: study design* |  |
| Theoretical framework |  |
| 9. Methodological orientation and Theory | The framework of the study were cognitive stress theories and surgical simulation-based training theories. The qualitative method used was qualitative content analysis adopted from Graneheim and Lundman. |
| Participant selection |  |
| 10. Sampling | Purposive sampling. We aimed for a group of surgical trainees which represented a realistic sample regarding previous laparoscopic simulation experiences. No participants withdrew from the study. |
| 11. Method of approach | The participants were approached by email and face-to-face by the first author. Participants received written information about the study and had the opportunity to ask questions ahead of the interview. |
| 12. Sample size | 20 participants were included in the final analysis. |
| 13. Non-participation | None of the included participants withdrew. |
| Setting |  |
| 14. Setting of data collection | The data were collected in a private office at the training facilities. |
| 15. Presence of non-participants | No one else was present besides the participants and the researcher MST. |
| 16. Description of sample | The sample consisted of surgical trainees with 1-3 years of specialist training. Demographics are presented in text and in Table 3. |
| Data collection |  |
| 17. Interview guide | The interview guide is provided as additional file 2. The guide was piloted. |
| 18. Repeat interviews | No repeated interviews were carried out. |
| 19. Audio/visual recording | All interviews were audio recorded and stored in accordance with recommendations of the regional ethics committee. |
| 20. Field notes | N/A |
| 21. Duration | The interviews lasted for 20-60 minutes. |
| 22. Data saturation | Data saturation was reached when no new themes were identified through preliminary analysis. |
| 23. Transcripts returned | Transcripts were not returned to participants; however, the interviews were summarized, and participant could orally comment and correct their statements. |
| *Domain 3: analysis and findings* |  |
| Data analysis |  |
| 24. Number of data coders | The main author coded the data in collaboration with a co-author |
| 25. Description of the coding tree | Themes were derived from data. No coding tree was used. |
| 26. Derivation of themes | Themes were derived from the data. |
| 27. Software | To manage data the method described by Ose was used. |
| 28. Participant checking | The participants did not provide feedback on the overall findings. However, at the end of the interviews, a summary of interview was given, and participants could comment and correct the interview contents. |
| Reporting |  |
| 29. Quotations presented | Themes are presented and illustrative quotations/excerpts are presented in Table 2. The excerpts are identified by participant number. |
| 30. Data and findings consistent | The presented data and findings are consistent. These are presented in Table 2. |
| 31. Clarity of major themes | Major themes are presented in results section and in Table 2. |
| 32. Clarity of minor themes | Minor themes are presented in results section and in Table 2. |
