# Supplementary material for: Simulation-based skills training: a qualitative interview study exploring surgical trainees’ experience of stress
Source: Adv Simul (Lond). 2022 Oct 22;7:33. doi: 10.1186/s41077-022-00231-2 (PMC9588224; doi:10.1186/s41077-022-00231-2)
Supplement: Supplementary file 2 — Additional file 2. Interview guide. Description of data: Topical questions used in the interviews for data collection. [file 41077_2022_231_MOESM2_ESM.docx]

**Interview guide - Subjective stress experiences in laparoscopic simulation-based training course**

**Introduction:**

**Background and previous experience**

We want to know if you experienced stress during the three days at this laparoscopic simulation-based training course, and we want to understand what factors and features of the simulation-based training course you perceived as stressful?

1. We would like to know about your background and your experiences regarding simulation, laparoscopy technique and practice.

*Probes:*

- *Date of birth*
- *Basic medical education, professional practice postgraduate, surgical discipline(s)*
- *Experience with simulation-based training (context, type, length of time and estimated hours spent, simulator modalities)*
- *Experience with laparoscopic surgery (**estimated number of laparoscopic operations observed, assisted, or performed)*
- *Experience with laparoscopic technique and skills (estimated number of hours spent training on simulators, type of laparoscopic simulators, robotic laparoscopic systems)*

1. We would like to know how you divide the time between work in clinical settings and the time you spend learning/training/performing laparoscopy techniques and skills?

*Probes:*

- *Estimated number of hours practicing laparoscopic technique and skill during a regular work week, month, or year.*

1. We would like to know if you have previous experience with computer games.

*Probes:*

- *Familiarity with computer-based gaming*
- *Familiarity with computer-based virtual reality interface*
- *Familiarity with computer-based gaming techniques*
- *Approximately amount of time spent as a player of computer games*
- *Estimated number of years*
- *Estimated hours per week*

1. We would like to know your experience with e.g., handwork, knitting, needlecrafts, playing string instruments or other activities that requires a high degree of motoric precision?

*Probes:*

- *Approximately number of years' experience?*
- *Hours per week?*

**Main topic:**

**Stress experiences in this laparoscopic SBT course (trainees overall experience)**

Please, describe your experiences of stress or stressors during this laparoscopic SBT course.

*Probes:*

*- The course set-up*

- *Stress related to technical demands*
- *Stress related to cognitive demands*
- *Stress related to time pressures*
- *Combination of technique, cognitive demands, and time pressure*
- *Pressures from instructors*
- *Pressures from course participants (training partners)*
- *Self-imposed pressure (personality trait, high /low stakes task, embodied surgeon role, concerns about transferability to real context)*
- *Influences of other participants or instructors*

1. Please, describe what external factors elicited your experience of stress during the SBT course?

*Probes:*

- *Noise*
- *Parallel conversations*
- *Ambient temperature*
- *Lighting conditions*
- *Your working postures*
- *Psychomotor demands*
- *Task requirements related*
- *Equipment related*

1. Please, describe what internal factors elicited your experience of stress during the SBT course?

*Probes:*

- *Personal ambitions*
- *Personality traits*
- *Learning habits*
- *Site-specific*
- *Situational-specific*
- *The collaborative relationships with your training partner*
- *Fear of making mistakes or errors*

1. How would you describe the influence of the stress experience had on the SBT course?

**Stress experiences related to simulators (trainees’ stress experiences related to each simulator)**

You have now been training on the simulator modalities the D-box, P.O.P-trainer and LapMentor™ through three days.

1. Please, describe if you experienced any stress while training on the simulators?

*Probes: why did/did you not experience stress?*

1. Please describe what stress experiences you had when training on the D-box, P.O.P-trainer and LapMentor™?
2. Why do you think you experienced stress at this/these modality/modalities (D-box, P.O.P-trainer and LapMentor™)?

*Probes:*

- *The simulation tasks for D-box, P.O.P- trainer and LapMentor™*
- *Stress related to the computer interface, software, other software related issues*
- *Stress related to the laparoscopic instruments and other equipment issues*
- *Stress related to technical demands* *of simulation tasks*
- *Stress related to cognitive demands* *of simulation tasks*
- *Stress related to time pressures* *of simulation tasks*
- *Combination of technique, cognitive demands, and time pressure*
- *Pressures from instructors*
- *Pressures from course participants (training partners)*
- *Internal pressures/subjective pressures*
- *Influences of other participants or instructors*

1. How would you describe the influence of the stress experience had on training and performance?

**Closure:**

Do you have any thoughts, perspectives, or related topics you would like to embellish or specify?

**Closing the interview:**

Make a recap of dominant interview themes, ask interviewee to verify or clarify their statements and views.
